# Epidemiology of Hypertensive State among Chinese Migrants: Effects of Unaffordable Medical Care

**DOI:** 10.1155/2018/5231048

**Published:** 2018-05-29

**Authors:** Ming Guan

**Affiliations:** ^1^Family Issues Center, Xuchang University, Road Bayi 88, Xuchang, Henan, China; ^2^School of Business, Xuchang University, Road Bayi 88, Xuchang, Henan, China

## Abstract

Hypertension is a major risk factor for heart disease and stroke. Affordability of medical care affects hypertension prevention, treatment, and control, but limited information is available for Chinese migrants with hypertensive state. Using Longitudinal Survey on Rural Urban Migration in China 2009 data, 2468 Chinese migrants reported hypertensive status. On the basis of comparison between medical payment and job income, participants were categorized as unaffordable and affordable. Thus, unaffordable expenses and unaffordable services were defined based on a public available survey. The descriptive statistics showed that 24.96% were at risk of prehypertension and mild-moderate-severe hypertension among 2468 Chinese migrants from 15 cities. Small part of the sample was not affordable to pay medical expenses and services. There were significant differences of hypertensive states between gender, marital status, regular smoker, and economic unaffordability. Multiple logistic regressions indicated that economic unaffordability had associations with abnormal weight, poor health assessment, and unhealthy hypertensive status. The alarming results may necessitate targeted interventions, even among people with good health status.

## 1. Introduction

Hypertension was an important public health burden in China, and control of hypertension was still suboptimal [1]. The trend of an increase in prevalence of hypertension in China was striking in young people and rural populations [2]. Hypertension prevalence in north China was 9.1% (95% CI: 4.1–14.1), which was higher than south China [3]. The treatment and control status of incident hypertension, while improved, remained very poor [4]. Rates of awareness and treatment of hypertension remained low and blood pressure was poorly controlled [5]. There was an urgent need for public health strategies with more emphasis on improvement of primary healthcare in China [6]. Inadequate counseling, lack of understanding on the disease, difficulties in accessing specialist care, and poor medication adherence were the barriers to optimal blood pressure control [7]. In addition, many cases of hypertension were going undetected and untreated under the effective health system in China [8]. Inadequate health insurance coverage for the high hypertension-risk migrants remained a challenge for the Chinese health reform [9].

Clinically, hypertension transits from normal blood pressure (normal pressure) to prehypertension, moderate hypertension, and severe hypertension. Normal pressure was defined as 90 mm Hg < systolic blood pressure < 120 mm Hg and 60 mm Hg < diastolic blood pressure < 80 mm Hg [10]. Prehypertension referred to a systemic BP of 120–139 mm Hg systolic and/or 80–89 mm Hg diastolic [11]. Prehypertension was often associated with other cardiovascular risk factors and independently increases the risk of hypertension and subsequent cardiovascular events [12]. Mild-and-moderate hypertension also was defined (mean sitting systolic BP/mean sitting diastolic BP, mild: 140–179/90–109 mmHg) [13]. Thus, severe hypertension was defined as (mean sitting systolic BP/mean sitting diastolic BP, severe: 180–/110– mmHg).

The primary care was effective in managing hypertension irrespective of management and operation models in urban China [14]. The migrants, the poor, and the vulnerable remained in the edge of the health insurance system [15]. Thus, subjects from rural migrants in urban China can be considered as a specifically medical and clinical targeted group.

The objective of this study was to assess the relationship between healthcare affordability and its association with change of hypertensive state among the Chinese migrants. Hypertensive state was defined under the combination of systolic pressure and diastolic pressure. Multiple logistic regressions were conducted to compute association of economic unaffordability with BMI, health assessment, and hypertensive states.

## 2. Methods

### 2.1. Data Source

This study used data from the Longitudinal Survey on Rural Urban Migration in China (RUMiC) organized by Professor Xin Meng at Australian National University. Detailed information is available at https://datasets.iza.org/. The core component was conducted in 15 Chinese cities (Guangzhou, Dongguan, Shenzhen, Zhengzhou, Luoyang, Hefei, Bengbu, Chongqing, Shanghai, Nanjing, Wuxi, Hangzhou, Ningbo, Wuhan, and Chengdu).

### 2.2. Main Variables

Unaffordability was defined based on the following questions: (a) Expenses: “how much were your total medical expenses on the disease in the last three months?” (b) Services: “what was the total amount of your cash expenditure on medical services in 2007?” (c) Income: “what is your average monthly income from current primary job?” Thus, two variables, “unaffordable expenses” and “unaffordable services,” were constructed after income minus the two types of expenditure, respectively. Subsequently, “unaffordable expenses” and “unaffordable services” were also dichotomized into 1 and 0, when they were above 0 and equal to or below 0. Negative values were defined as unaffordability, while zero and positive values were defined as affordability.

Sociodemographic characteristics collected were age (9–25, 26–40, and ≥41 years), sex, marital status (married and other), current work status (employed and other), and BMI. Obesity was defined using BMI according to the Chinese criteria: underweight (less than 18.5 kg/m^2^), normal (18.5–23.9 kg/m^2^), overweight (24.0–27.9 kg/m^2^), and obese (equal to or more than 28.0 kg/m^2^) [16].

### 2.3. Statistical Analysis

The distributions of normal pressure, prehypertension, and mild-moderate-severe (MMS) hypertension were assessed by sociodemographic characteristics. Using unaffordable expenses and services as covariates, logistic regression models were mainly used to assess the associations of hypertensive state, while adjusting for age, sex, marital status, current work status, and education. All analyses accounted for the RUMiC complex sample design by using STATA 14.0 for Windows (STATA Corp, College Station, TX, USA).

## 3. Results

Overall, 63.25% of the sample was males among 2468 individuals aged ≥18 years included in the study. The participants had the mean age of 32.37 years (95% CI 31.26–33.46, range 18–72 years), with mean systolic blood pressure of 119.22 mm Hg (95% CI 118.78–119.66, range 90.01–191.67 mm Hg), and mean diastolic blood pressure of 75.90 mm Hg (95% CI 75.58–76.22, range 60.02–124.67 mm Hg).

In Table 1, there were significant differences of hypertensive state among age, gender, marital status, regular smoker, unaffordable expenses, and unaffordable services. Among the 2468 participants, 19.85% was risk of prehypertension, and 5.11% was risk of MMS hypertension. 1.30% was unaffordable to pay medical expenses. 8.47% was unaffordable to pay medical services. The migrants who could not afford medical services were more than migrants who could not afford medical expenses.

In Table 2, unaffordable services were significantly associated with being underweight, overweight, and obese. But, the odds ratios were below 1. In Table 3, unaffordable expenses were significantly associated with average and poor health assessment. Unaffordable services were associated with excellent, good, average, poor, and very poor health assessment. But, the odds ratios were below 1. This suggested that increase in unaffordable services would produce decrease in dimensions in health assessment.

In Table 4, age range 26–40 was associated with normal pressure (AOR: 1.380; 95 CI: 1.150–1.657), prehypertension (AOR: 0.598; 95 CI: 0.494–0.723), and MMS hypertension (AOR: 0.097; 95 CI: 0.069–0.137). Age ≥41 was associated with prehypertension (AOR: 0.560; 95 CI: 0.428–0.732) and MMS hypertension (AOR: 0.418; 95 CI: 0.310–0.565). This suggested that young migrants would be more likely to be in risk of hypertension than old migrants did. Female was associated with normal pressure (AOR: 3.823; 95 CI: 3.059–4.777), prehypertension (AOR: 0.263; 95 CI: 0.207–0.335), and MMS hypertension (AOR: 0.206; 95 CI: 0.140–0.301). Never married status was associated with normal pressure (AOR: 3.519; 95 CI: 2.865–4.323), prehypertension (AOR: 0.266; 95 CI: 0.214–0.330), and MMS hypertension (AOR: 0.034; 95 CI: 0.019–0.060). Regular smoker was associated with normal pressure (AOR: 1.258; 95 CI: 1.012–1.564), prehypertension (AOR: 0.780; 95 CI: 0.621–0.978), and MMS hypertension (AOR: 0.474; 95 CI: 0.331–0.678). Unaffordable services were associated with normal pressure (AOR: 0.659; 95 CI: 0.462–0.940) and prehypertension (AOR: 1.672; 95 CI: 1.153–2.425). This suggested that female, never married, regular smokers with higher age were not likely to be at risk of prehypertension and MMS hypertension. Persons with unaffordable services were likely to be at risk of prehypertension. Due to the smallest odds ratio in the table, never married subjects face the highest risk of hypertension.

## 4. Discussion

The findings of this study indicate that hypertensive state has been very prevalent in Chinese migrants. Part of Chinese migrants suffering from hypertension was accompanied by unaffordable expenses and services. With respect to high medical fees, affordable coverage could not be available to each migrant. Even worse, this affliction falls particularly hard on low-wage workers due to high cost of medical services. Thus, Chinese migrants with hypertension were less likely to report affordable medical expenses and services which produced inadequate healthcare access. This study suggested that, even among those who afforded medical expenses and services, barriers to receiving affordable healthcare were still a challenge.

A possible explanation for the unaffordable expenses was due to lack of basic health insurance which dramatically increased the risk of depression based on northwestern Chinese community samples [17]. It is a common sense that mental disorders are risk factors for hypertension. China has achieved high population coverage rate over a short time period, starting with a limited benefit package. However, poor people have less benefit from New Cooperative Medical Scheme (NCMS) in terms of health service utilization [18]. The prices, availability, and affordability of medicines in China were not equitable access to basic medical treatments, especially for the poor [19]. Thus, psychiatric disorders produced by socioeconomic factors increased the risk of hypertension, when the migrants were out of the protection of social, health, and medical insurance.

Unaffordable medical care can be explained by marketization of health and social service organizations. The finding that a significantly higher proportion of married, employed, and women migrants aged 36 to 44 years had unaffordable medical care suggested that factors other than individual (such as employer) were also related to insurance status. Moreover, the insured still paid high amount of out-of-pocket medical expenditure [20]. NCMS improved the situation of receiving healthcare services but did not reduce the high healthcare fees [21]. The rapid increase of public funding to subsidize health insurance in China did not mitigate the out-of-pocket payment for healthcare over the past decade [22]. Thus, organizational, institutional, and employers' power dominate affordability, accessibility, and availability of Chinese migrants' healthcare.

This finding was consistent with those of earlier studies. For example, a study concluded that rural-urban migrant workers might well be left out of sharing the social and economic development [23]. Another study indicated that young migrant male workers appeared to be most vulnerable in their psychological well-being [24]. A published literature also argued that health systems governance was criticized as ineffective and inefficient in the development and operation of Cooperative Medical Scheme and New Rural Cooperative Medical Scheme [25]. Thereby, poor connection between urban and rural insurance system was partially responsible for the omission of the migrants' health support plans.

Together, these findings show that the healthcare system should seek to reduce the barriers for these patients to achieve BP control. Enrolled in the Urban Employee Basic Health Insurance scheme, people with hypertension in Shanghai, China, had no access to publicity of hypertension prevention knowledge [26]. For the floating population, health insurance coverage needs to be improved [27]. The fragmented health insurance schemes generated inequitable healthcare utilization and health outcomes for the elderly [28]. In order to effectively control hypertension, a study highlighted better health insurance packages [29]. These findings may encourage employment service agencies to expand the coverage of health service from a specific targeted population to general population included migrants.

This finding was in line with the previous research that unaffordable expenses, unaffordable insurance, and unaffordable services were related to income inequality. Based on the China Health and Nutrition Survey (1991–2006), health disparities in China were related to rising income inequality and in particular to the adverse health and income experience of older (wo)men [30]. Based on US data from four waves of the Panel Study of Income Dynamics, low wages were risk factors for hypertension among working people for women and persons aged 25–44 years [31]. Comparing 2009 with 2006, the income inequality in health insurance coverage was largely corrected in China through rapid expansion in rural areas and initiation of urban resident basic medical insurance in urban areas [32]. This combination of findings provided some support for the intuition that income inequality caused by poor distribution of wealth resources led to health vulnerabilities of the marginalized persons.

This study had several limitations. First, all hypertension information collected from the RUMiC was from measurement of systolic pressure and diastolic pressure. In fact, most of the Chinese migrants with hypertension were unaware of the condition, leading to under-reporting of barriers to care in this population. In addition, Chinese migrants with hypertension were unaware of blood control. Second, the RUMiC did not provide information on antihypertensive care and cure. Thus, the expenditure of antihypertensive cure and hypertension affordability could not be assessed and estimated scientifically. Third, the survey provided no information about the affordable medical expenditure as a proportion of household income. Thus, a method of more accurate unaffordable measure could not be conducted. Finally, given that only 15 cities were surveyed, the data may not be nationally representative.

## 5. Conclusion

In conclusion, the hypertensive states were serious in the Chinese migrants. There were significant differences between hypertensive states among the migrants with respect to affordable and unaffordable medical expenses and services. Also, part of Chinese migrants with hypertension was likely to report unaffordable care under the condition that there were significant differences between hypertensive states among the migrants with respect to specific sociodemographic factors. Among the Chinese migrants, economic unaffordability was associated with abnormal weight, poor health status, and hypertensive states. Subsequently, some policies and interventions suggested were highlighted.

## Figures and Tables

**Table 1:** Demographic characteristics among adults with hypertensive state, *N* (%).

	Normal pressure	Prehypertension	MMS hypertension	chi2	*p* value
Age				207.6162^*∗∗∗*^	0.000
9–25	756 (30.63)	111 (4.50)	12 (0.49)		
26–40	829 (33.59)	258 (10.45)	40 (1.62)		
≥41	267 (10.82)	121 (4.90)	74 (3.00)		
Gender				98.7121^*∗∗∗*^	0.000
Male	973 (39.42)	370 (14.99)	94 (3.81)		
Female	879 (35.62)	120 (4.86)	32 (1.30)		
Marital status				126.4450^*∗∗∗*^	0.000
Married	967 (39.18)	346 (14.02)	104 (4.21)		
Remarried	14 (0.57)	4 (0.16)	2 (0.08)		
Cohabited	21 (0.85)	0 (0.00)	0 (0.00)		
Divorced	32 (1.30)	11 (0.45)	2 (0.08)		
Widowed	9 (0.36)	5 (0.20)	5 (0.20)		
Never married	809 (32.78)	124 (5.02)	13 (0.53)		
Physical disabilities				0.9726	0.615
No	1,781 (72.16)	467 (18.92)	122 (4.94)		
Yes	71 (2.88)	23 (0.93)	4 (0.16)		
Sick injured				2.5501	0.279
No	1,514 (61.35)	395 (16.00)	96 (3.89)		
Yes	338 (13.70)	95 (3.85)	30 (1.22)		
Regular smoker				33.5242^*∗∗∗*^	0.000
No	1,369 (55.47)	303 (12.28)	77 (3.12)		
Yes	483 (19.57)	187 (7.58)	49 (1.99)		
Unaffordable expenses				8.3172^*∗∗*^	0.016
No	1,835 (74.35)	478 (19.37)	123 (4.98)		
Yes	17 (0.69)	12 (0.49)	3 (0.12)		
Unaffordable services				12.7682^*∗∗∗*^	0.002
No	1,715 (69.49)	429 (17.38)	115 (4.66)		
Yes	137 (5.55)	61 (2.47)	11 (0.45)		

^*∗∗*^ *p* < 0.05; ^*∗∗∗*^*p* < 0.01.

**Table 2:** Association between BMI and economic unaffordability.

	Underweight		Normal weight		Overweight		Obese	
	AOR	95 CI	AOR	95 CI	AOR	95 CI	AOR	95 CI
Unaffordable expenses	0.358	0.074–1.734	1.361	0.631–2.935	0.891	0.366–2.166	0.577	0.102–3.260
Unaffordable services	0.144^*∗∗∗*^	0.093–0.222	1.242	0.927–1.664	0.345^*∗∗∗*^	0.248–0.481	0.077^*∗∗∗*^	0.044–0.132

^*∗∗∗*^ *p* < 0.01.

**Table 3:** Association between health assessment and economic unaffordability.

	Excellent		Good		Average		Poor		Very poor	
	AOR	95 CI	AOR	95 CI	AOR	95 CI	AOR	95 CI	AOR	95 CI
Unaffordable expenses	0.391	0.128–1.193	1.190	0.557–2.544	0.344^*∗*^	0.126–0.939	3.470^*∗*^	1.207–9.974	1.503	0.120–18.892
Unaffordable services	0.331^*∗∗∗*^	0.237–0.463	0.480^*∗∗∗*^	0.352–0.656	0.511^*∗∗∗*^	0.376–0.694	0.095^*∗∗∗*^	0.057–0.158	0.018^*∗∗∗*^	0.007–0.045

^*∗*^0.05 < *p* < 0.10; ^*∗∗∗*^*p* < 0.01.

**Table 4:** Association between hypertensive states with economic unaffordability.

	Normal pressure	Prehypertension	MMS hypertension
	AOR (95% CI)	AOR (95% CI)	AOR (95% CI)
Age			
9–25	1.00 (reference)	1.00 (reference)	1.00 (reference)
26–40	1.380^*∗∗∗*^ (1.150–1.657)	0.598^*∗∗∗*^ (0.494–0.723)	0.097^*∗∗∗*^ (0.069–0.137)
≥41	0.847 (0.660–1.086)	0.560^*∗∗∗*^ (0.428–0.732)	0.418^*∗∗∗*^ (0.310–0.565)
Gender			
Male	1.00 (reference)	1.00 (reference)	1.00 (reference)
Female	3.823^*∗∗∗*^ (3.059–4.777)	0.263^*∗∗∗*^ (0.207–0.335)	0.206^*∗∗∗*^ (0.140–0.301)
Marital status			
Married	1.00 (reference)	1.00 (reference)	1.00 (reference)
Remarried	0.874 (0.301–2.542)	0.918 (0.286–2.945)	1.586 (0.325–7.747)
Divorced	1.103 (0.574–2.121)	1.028 (0.535–1.974)	0.430 (0.085–2.169)
Widowed	0.473 (0.162–1.378)	1.226 (0.379–3.968)	2.589 (0.740–9.052)
Never married	3.519^*∗∗∗*^ (2.865–4.323)	0.266^*∗∗∗*^ (0.214–0.330)	0.034^*∗∗∗*^ (0.019–0.060)
Physical disabilities			
No	1.00 (reference)	1.00 (reference)	1.00 (reference)
Yes	1.098 (0.677–1.781)	1.007 (0.605–1.676)	0.472 (0.173–1.290)
Sick injured			
No	1.00 (reference)	1.00 (reference)	1.00 (reference)
Yes	1.049 (0.810–1.358)	0.861 (0.651–1.138)	0.997 (0.620–1.602)
Regular Smokers			
No	1.00 (reference)	1.00 (reference)	1.00 (reference)
Yes	1.258^*∗*^ (1.012–1.564)	0.780^*∗*^ (0.621–0.978)	0.474^*∗∗∗*^ (0.331–0.678)
Unaffordable expenses			
No	1.00 (reference)	1.00 (reference)	1.00 (reference)
Yes	0.595 (0.246–1.436)	1.710 (0.703–4.156)	1.800 (0.402–8.064)
Unaffordable services			
No	1.00 (reference)	1.00 (reference)	1.00 (reference)
Yes	0.659^*∗*^ (0.462–0.940)	1.672^*∗∗*^ (1.153–2.425)	0.588 (0.274–1.263)

^*∗*^ *p* < 0.10; ^*∗∗*^*p* < 0.05; ^*∗∗∗*^*p* < 0.01.

## Data Availability

The 2009 RUMiC data used to support the findings of this study are available at http://idsc.iza.org/rumic.
